# Dirofilariosis in the Americas: a more virulent *Dirofilaria immitis*?

**DOI:** 10.1186/1756-3305-6-288

**Published:** 2013-10-02

**Authors:** Filipe Dantas-Torres, Domenico Otranto

**Affiliations:** 1 Department of Immunology, Aggeu Magalhães Research Centre, Oswaldo Cruz Foundation, Recife, Pernambuco 50670420, Brazil; 2 Department of Veterinary Medicine, Faculty of Veterinary Medicine, University of Bari, Valenzano, Bari 70010, Italy

**Keywords:** *Dirofilaria immitis*, Americas, Genetics, Pathogenicity, Virulence, Zoonosis

## Abstract

Dirofilarioses are widespread diseases caused by filarioid nematodes (superfamily Filarioidea) of the genus *Dirofilaria*, which are transmitted by a plethora of mosquito species. The principal agent of canine dirofilariosis in the Americas is *Dirofilaria immitis*, which may also occasionally infest humans, resulting in pulmonary nodules that may be confounded with malignant lung tumours. Because human cases of dirofilariosis by *D. immitis* are relatively frequent in the Americas and rare in Europe and other eastern countries, where *Dirofilaria repens* is the main causative agent, the existence of a more virulent strain of *D. immitis* in the Americas has been speculated. Recently, a case of human ocular infestation by *Dirofilaria* sp*.* was diagnosed in Pará State, northern Brazil, where canine heartworm dirofilariosis is endemic. The nematode was shown to be morphologically and phylogenetically related to *D. immitis* but it was genetically distinct from reference sequences, including those of *D. immitis* infesting dogs in the same geographical area. This finding raised questions regarding the aetiology of human dirofilariosis in the Americas, since information on the genetic makeup of filarioids infesting dogs and humans is meagre. Further studies would be needed to better characterize filarioids infesting dogs, wild animals, and humans in the Americas and to assess the existence of a more virulent *D. immitis* strain in this continent. Finally, the competence of different culicid species/strains from Europe and the Americas as vectors of *Dirofilaria* species should be investigated. Such studies would help us to understand possible variations in transmission patterns and even to predict possible scenarios that may emerge in the future, with the introduction of non-endemic *Dirofilaria* species/strains in free areas through importation of infested animals, vectors, or both.

## Introduction

Dirofilariosis is a worldwide-distributed disease caused by nematodes of the genus *Dirofilaria* of the family Onchocercidae. These nematodes may infest wild and domestic mammals of several orders, such as Artiodactyla, Carnivora, Edentata, Lagomorpha, Perissodactyla, Primates, and Rodentia [1,2]. Most of the infested animals display no apparent clinical signs or laboratory abnormalities. However, some animals may develop clinical disease, which may range from benign, localized subcutaneous nodules to life-threatening, systemic conditions. For example, dogs infested by *Dirofilaria immitis*, also known as heartworm, can present with respiratory distress, epistaxis, haemoptysis, ascites, exercise intolerance, and anorexia [3,4]. Importantly, the treatment of canine dirofilariosis by *D. immitis* can be expensive and often associated with life-threatening complications, particularly in patients with moderate to severe heartworm disease [5].

Even if canine dirofilariosis is still widespread and highly prevalent worldwide, the availability of preventatives, improved diagnostic tools and different treatment options [3,4] have greatly contributed in reducing the number of severe clinical cases, particularly those with a fatal outcome. Nonetheless, *Dirofilaria* infestations in dogs are still of major veterinary and public health concern, considering that canine and human dirofilariosis continue to be diagnosed in several tropical, subtropical and temperate regions of the world [4,6,7].

Human cases of dirofilariosis have been reported worldwide. In the Old World, most cases refer to subcutaneous infestations by *Dirofilaria repens*, whereas in the New World pulmonary dirofilariosis by *D. immitis* predominates [3,4]. Nonetheless, it has been acknowledged that the aetiology of several human cases remains doubtful because the species identification is usually based on histological findings only. For example, a review of 28 cases of human dirofilariosis attributed to *D. immitis* or to a species other than *D. repens* in the Old World put in doubt the pathogenic role of the former species in humans in this region [8]. Indeed, even if the occurrence of human dirofilariosis by *D. immitis* in Europe has been ascertained [9,10], the great majority of human pulmonary and subcutaneous infestations in the Old World are associated with *D. repens*[4]; except in Japan, where *D. immitis* prevails [11]. Although the zoonotic potential of different *Dirofilaria* species is well recognized, the control and prevention of *Dirofilaria* infestations in reservoir hosts is often neglected.

Information on the aetiology and, thus, eco-epidemiology of animal and human dirofilariosis in the Americas is fragmentary, particularly in South America. For instance, a recent human case of dirofilariosis from northern Brazil was attributed to a nematode morphologically and phylogenetically close to *D. immitis* but genetically distinct from reference sequences, including those of *D. immitis* infesting dogs in the same geographical area [12]. These findings raise interesting questions regarding the *Dirofilaria* species infesting wild and domestic animals in South America, as well on the aetiology of human pulmonary and subcutaneous dirofilariosis in the same region. Within this context, the present review focuses on some aspects related to the dirofilariosis and *Dirofilaria* parasites in the Americas, with an emphasis on South America, and lists future research needs on this neglected field of human parasitology.

## Review

### Diversity of dirofilariae

Dirofilariae are spirurid nematodes, which localize, with a few exceptions, in subcutaneous tissues of mammalian hosts (e.g., foxes, coyotes, wolves, dogs, sea lions, harbour seals, ferrets, horses, bears, wolverines, muskrats, raccoons, bobcats, cats, monkeys, and red pandas) and are transmitted predominately by mosquitoes [1,2,13-25]. The genus *Dirofilaria* consists of 27 apparently valid species (Table 1) and 15 species of questionable validity [1]. Moreover, 10 additional species of *Dirofilaria* have been replaced into other genera [1]. Potentially new species have also been proposed (e.g., “*Candidatus* Dirofilaria hongkongensis”) [24], but considering the current number of doubtful species within this genus [1,26] any description of a new species at this stage, mainly if supported only by genetic data, would be premature. In addition, a revision of the genus *Dirofilaria* based on robust morphological and genetic data would be needed before the description of any new species.

**Table 1:** **The genus *Dirofilaria***

**Subgenus and species**	**Host (families)**	**Geographical distribution**
Subgenus *Dirofilaria*		
*D. ailure* Ryjikov and Románova, 1961	Procyonidae	China
*D. freitasi* Machado de Mendonca, 1949	Bradypodidae	Brazil
*D. immitis* (Leidy, 1856)	Canidae, Felidae, Hominidae, and many others	Cosmopolitan
*D. lutrae* Orihel, 1965	Mustelidae	USA
*D. spectans* Freitas and Lent, 1949	Hominidae (single case), Mustelidae	Brazil
Subgenus *Nochtiella*		
*D. acutiuscula* (Molin, 1858)	Canidae, Caviidae, Felidae, Tayassuidae	South America, USA
*D. bonnie* Vogel and Vogelsang, 1930	Muridae	Java
*D. cancrivori* Eberhard, 1978	Procyonidae	Guyana
*D. corynodes* (Linstow, 1899)	Cercopithecidae	Africa, Thailand
*D. genettae* Baylis, 1928	Felidae, Viverridae	Nigeria
*D. granulosa* (Linstow, 1906)	Felidae	Africa, Asia
*D. incrassata* (Molin, 1858)	Bradypodidae, Procyonidae	Brazil and Central America
*D. linstowi* Dissanaike, 1972	Cercopithecidae	Sri Lanka
*D. macacae* Sandground, 1933	Cercopithecidae	Indochina
*D. macrodemos* Eberhard, 1978	Bradypodidae	Guyana, Panama
*D. magnilarvata* Price, 1959	Cercopithecidae, Hominidae, Hylobatidae	Malaya
*D. minor* Sandground, 1933	Felidae	Vietnam
*D. pagumae* Sandground, 1933	Viverridae	Indochina
*D. panamensis* Eberhard, 1978	Bradypodidae	Panama
*D. repens* Railliet and Henry, 1911	Canidae, Felidae, Hominidae, Viverridae	Europe, Asia, Africa
*D. sachsi* Shoho, 1974	Bovidae	East Africa
*D. striata* (Molin, 1858)	Canidae, Felidae, Hominidae (single case), Tayassuidae	Brazil, Venezuela, USA
*D. subdermata* Mönnig, 1924	Erethizontidae	North America, South Africa
*D. sudanensis* (Linstow in Schipley 1902)	Felidae, Hyaenidae	Sudan
*D. tawila* Khalil, 1932	Cercopithecidae	Africa
*D. tenuis* Chandler, 1942	Hominidae, Procyonidae	North America
*D. ursi* Yamaguti, 1941	Felidae, Hominidae, Ursidae	Asia, North America

Checklist of valid species of *Dirofilaria* (adapted from Ref. [1]). Species of questionable validity are not listed.

In the Americas, several *Dirofilaria* species have been reported from wild and domestic mammals. In Brazil alone, eight *Dirofilaria* species – i.e., *D. acutiuscula*, *D. freitasi*, *D. incrassata*, *D. immitis*, *D. magalhaesi*, *D. repens*, *D. spectans*, and *D. striata* – have been reported so far [2], even if the validity of at least one of those species (i.e., *D. magalhaesi*) has been questioned [1]. Moreover, the presence of *D. repens* in this country, and in the Americas as a whole, remains doubtful [4].

The presence of *D. repens* in the Americas has been first reported in a dog from São Paulo, south-eastern Brazil [26]. Recently, microfilariae resembling those of *D. repens* were detected in dogs from a semi-rural district near Santiago (Chile), but these microfilariae were larger and genetically distinct from *D. repens*[27]. In any case, these findings indicate that the diversity of *Dirofilaria* species in the Americas needs to be further investigated, also to determine whether cases of *D. repens* in dogs are being actually misdiagnosed as *D. immitis* based on the retrieval of blood circulating microfilariae.

### Mosquito vectors

Given the fragmentary data on *Dirofilaria* species infesting wild and domestic animals in the Americas as well as the scant number of surveys on the mosquito species acting as potential vectors, the diversity of culicids transmitting *Dirofilaria* species in this region is currently underestimated. Indeed, little is known regarding the mosquito vectors of wildlife-associated *Dirofilaria* species, even considering that some of them (e.g., *D. spectans*, *D. tenuis*, and *D. ursi*) are of zoonotic concern [4]. On the other hand, several studies have succeeded in demonstrating the presence of infective third-stage larvae (L3) of *D. immitis* in naturally caught mosquitoes [28-31] or in proving experimentally the suitability of different mosquito species as proper intermediate hosts of this parasite [32-36].

*Dirofilaria immitis* can be transmitted by mosquitoes belonging to different genera, such as *Aedes* (*Ae*.), *Anopheles* (*An*.), *Culex* (*Cx*.), and *Ochlerotatus* (*Oc*.). For instance, a study conducted in Rio de Janeiro State, south-eastern Brazil, using canine, feline and human baits, reported *Oc. taeniorhynchus*, *Cx. quinquefasciatus*, *Oc. scapularis*, *Cx. declarator*, and *Cx. nigripalpus* as the most likely vectors of *D. immitis* in this region [37]. Indeed, *D. immitis* developmental stages were found in *Oc. scapularis*, *Oc. taeniorhynchus*, *Cx. quinquefasciatus*, *Cx. declarator*, *Cx. saltanensis* and *Wyeomyia bourrouli,* with L3 being found only in the first three species [28]. In another study conducted in Maranhão State, north-eastern Brazil, L3 were detected in *Cx. quinquefasciatus*[29]. Indeed, a subsequent experimental study demonstrated that *Cx. quinquefasciatus* supports the development of *D. immitis* to the L3 [34]. Similarly, non-infective stages of *D. immitis* were found in *Cx. pipiens* and *Stegomyia aegypti* and they have been regarded as putative vectors of this nematode in Argentina [38]. As a corollary, an experimental study conducted in Brazil confirmed the susceptibility of *St. aegypti* to *D. immitis*[36]. Meanwhile, studies conducted in the United States implicated several mosquito species as potential vectors of *D. immitis*, including *St. aegypti, Stegomyia albopicta*, *Oc. canadensis*, *Jarnellius sierrensis, Oc. trivittatus, Aedimorphus vexans*, *An. punctipennis*, *An. quadrimaculatus*, and *Cx. quinquefasciatus*[30,39-41]. Altogether, these studies underline that a plethora of mosquito species may act as vectors of *D. immitis* throughout the American continent, as it occurs in the Old World [42].

Worth mentioning, an interesting exception regarding *Dirofilaria* transmission is *D. ursi*, which infests American black bears (*Ursus americanus*) and is vectored by black flies (Simuliidae) [43].

### Transmission patterns

The American continent is extremely variegated in terms of topography, hydrography and climate. As such, the transmission patterns of different vector-borne pathogens may vary widely throughout this vast land territory. Still, the knowledge on the transmission patterns of *D. immitis* and other filarial nematodes in the Americas remains fragmentary. Some mosquito species may present high specificity for city regions (e.g., urban, suburban, and rural) and landscape elements within these regions (e.g., forest, housing density) [44]. For instance, a study in Puerto Rico showed the association of *St. aegypti* with high-density housing in urban areas, of *Cx. quinquefasciatus* with low-density housing in suburbs, and of *Gymnometopa mediovittata* and other native mosquitoes (*Cx. antillummagnorum*, *Toxorhynchites portoricencis*) with less disturbed habitats (forests, low-density housing) [44]. Therefore, the transmission of *D. immitis* may vary according to city region and landscape type. Indeed, studies indicate that the prevalence of heartworm infestation in dogs is usually higher in some coastal regions [45]. Because the development of mosquitoes is water and temperature dependent, wetlands (e.g., marsh, swamp, bog) and river valleys provide suitable environmental conditions for the vectors to develop, particularly in tropical and subtropical regions, where potential vectors of *D. immitis* are widespread and may be present throughout the entire year. For example, an entomological survey carried out in Rio de Janeiro, south-eastern Brazil revealed that *St. aegypti* and *C. quinquefasciatus* were present the year-round [46]. Accordingly, in these areas, the recommendation of preventatives (e.g., mosquito repellents and microfilaricides) becomes even more important towards the control of *D. immitis* infestation at the individual and population level.

In temperate regions, the presence of *D. immitis* vectors may be restricted to particular months of the year. For instance, mathematical models suggested that *D. immitis* transmission in Argentina is markedly seasonal (with peaks in January and February) and that no region of this country would support transmission throughout the year [47]. Undoubtedly, this sort of information may be valuable for veterinarians to recommend preventive strategies against *D. immitis* infestation in dogs, especially during high-risk months. The elaboration of optimized control strategies is particularly important in developing countries, where dog owners cannot always afford the costs of preventative chemoprophylactic measures.

### Canine and feline dirofilarioses

In the Americas, there are reports of infestation by *D. acutiuscula*, *D. immitis*, *D. repens*, and *D. striata* in dogs [1,2,6,45,48,49]. Undoubtedly, *D. immitis* is the most important causative agent of canine dirofilariosis [6,45], being found in most countries of the Americas, except Chile, French Guiana and Uruguay [4]. In the United States the infestation prevalence rate has been estimated to range from 1 to 12% [50]. In Central and South America, the prevalence rates may be much higher, reaching 42% in cities on the Gulf Coast of Mexico, 63.2% in the Caribbean (the Bahamas, Curaçao, Cuba, the Dominican Republic, and Puerto Rico), 45% in Brazil, and 74% in Argentina [4,6,38,45,51,52].

The reports of *D. acutiuscula* in a dog from Argentina [53]*, D. striata* in a dog from the United States [48], and *D. repens* in a dog from Brazil [26] and in dogs from Chile [27] are doubtful or need confirmation. Anyway, these findings raise some questions concerning the aetiology of canine dirofilariosis in the Americas as a whole. Indeed, a high nucleotide difference (5%) was found between 12S rDNA sequences generated from Chilean samples and a *D. repens* European sequence (GenBank accession number: AM779775), suggesting that the parasite found in Chile [27] may not be *D. repens*. Another study conducted in Marajó Island, northern Brazil, revealed some level of intra-specific difference in 5.8S and ITS2 regions [54]. In general, there is limited information on the genetic variability of filarioids infesting dogs worldwide. Certainly, further studies are urgently needed to obtain a more reliable picture regarding the species or genetic variants of dirofilariae circulating among dogs in the Americas.

In the Americas, feline dirofilariosis has been reported in the United States, Canada, Brazil, and Venezuela [4]. In a study carried out in northern Florida, necropsies performed on 630 adult cats revealed the presence of heartworms in 4.9% of them, with serological evidence of heartworm exposure in 17% of the tested population [55]. The highest rates of infection in cats parallel the levels of endemicity in dogs [4]. However, some studies in the United States and Brazil reported lower levels of exposure in cats living in areas were canine dirofilariosis is endemic [51], suggesting that the risk of infestation by *D. immitis* in cats may vary regardless of the prevalence of infestation in dogs from the same area.

### Human dirofilariosis in the Americas

**Figure 1:**
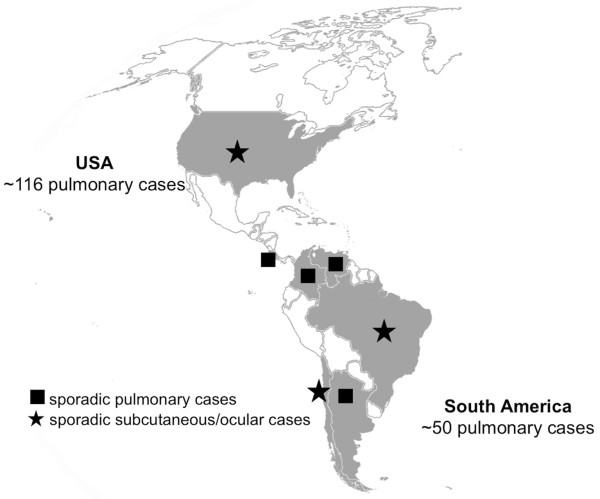
**Human dirofilariosis in the Americas.** Geographical distribution of human cases of dirofilariosis in the Americas (adapted from Ref. [4]). Countries in which *Dirofilaria immitis* cases predominate are in grey.

While most cases of human pulmonary dirofilariosis in the Americas have been attributed to *D. immitis*, other species may also infest and cause disease in humans in this region (Figure 1). For example, subcutaneous dirofilariosis in North America have been frequently attributed to *D. tenuis* and *D. ursi* (reviewed in Ref. [4]), which are primarily parasites of raccoons and bears, respectively [1]. *Dirofilaria spectans*, a parasite of otters in Brazil, has also been found in the digital artery of a human patient from Rio de Janeiro [56]. Similarly, *D. striata* (primarily found on wild felids) has been reported once in the orbit of a 6-year-old boy living in North Carolina [57]. Even considering that some species of *Dirofilaria* have been reported only once in humans, these case reports suggest that the number of potentially zoonotic dirofilariae in the American continent may be currently underestimated.

Little is known regarding the epidemiology and risk factors of human dirofilariosis in the Americas. The risk of infestation by some *Dirofilaria* species (e.g., *D. tenuis*) in humans is reputed to be higher in areas where there is a high incidence of these worms in their natural hosts [58]. Importantly, following natural disasters, free-roaming dogs may represent a public health risk since they may increase transmission of some significant diseases such as rabies, leptospirosis, Chagas disease, leishmaniasis and even dirofilariosis. Indeed, natural catastrophic events may cause mass migration of people, and animals with rehabilitation of displaced people in temporary human settlements under unhygienic conditions or relocation of animals. Latin America is second in terms of number of natural disasters only to Asia [59], and thus represents an area at risk for spreading of *Dirofilaria* species as a consequence of the fact that these nematodes may adapt to new animal hosts and arthropod vectors. At some specific occasions, such as in the case of hurricane Katrina in the United States, dogs infected by *D. immitis* were relocated from the areas stricken by the hurricane (e.g., Louisiana) to other states of North America, resulting in the introduction of this filarioid into previously non-endemic areas [60].

Most human cases of dirofilariosis reported in the international literature refer to subcutaneous/ocular dirofilariosis cases caused by *D. repens*[4]. The great majority of these cases come from the Old World, where *D. immitis* and *D. repens* may co-infest the same reservoir hosts [7]. In the Americas, human pulmonary dirofilariosis predominates and it is primarily associated with *D. immitis*. For instance, over 100 cases have been detected in the United States, most of which coming from south-eastern regions where *D. immitis* infestation in dogs is highly prevalent [4,50,61]. Cases of human pulmonary dirofilariosis have also been reported in Brazil [38,62,63] and, more rarely, in Costa Rica, Argentina, Venezuela, and Colombia [38,64-66]. Remarkably, approximately 70% of the cases reported in South America originated from south-eastern Brazil, particularly, from São Paulo city [67], one of the biggest medical poles in Latin America. Because the prevalence of *D. immitis* in dogs in São Paulo is low [68], the apparently high number of human pulmonary dirofilariosis from this state is probably due to the high standards of health care services provided. Indeed, there is no eco-epidemiological factor that could explain a higher risk of infestation in São Paulo city as compared with other Brazilian cities where canine dirofilariosis is endemic [49].

**Figure 2:**
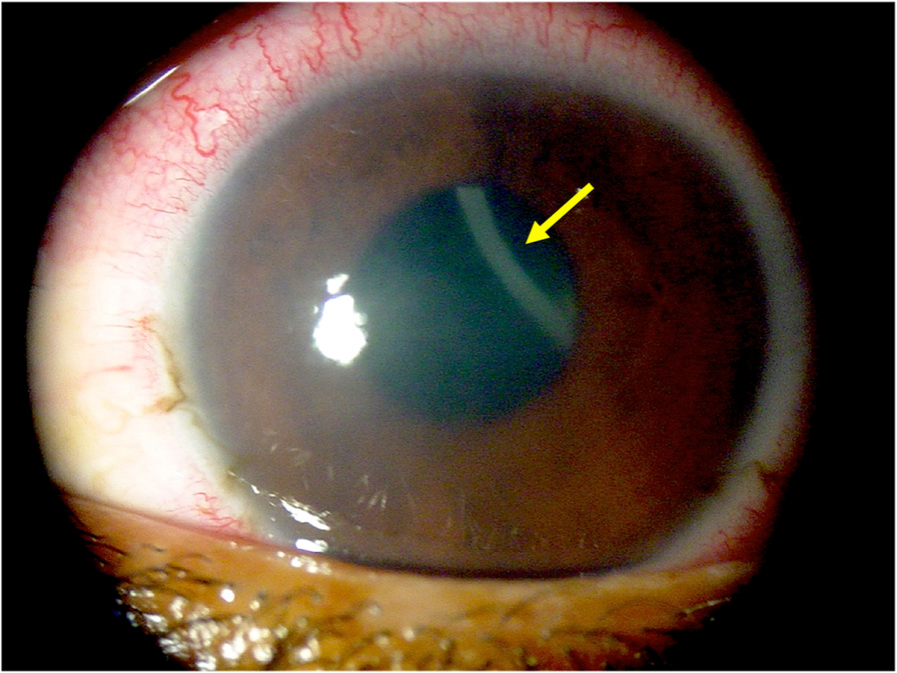
**Human ocular dirofilariosis in Brazil.** Corneal oedema and episcleral hyperaemia in the left eye of a 16-year-old boy from Brazil and a free-swimming filarioid (arrow) in the anterior chamber (adapted from Ref. [12]).

Subcutaneous/ocular dirofilariosis in the Americas have been attributed to different species, such as *D. tenuis*, *D. ursi* and *D. immitis* (a single case) in North America [4,61]. In addition, two interesting cases of subcutaneous and ocular dirofilarioses in human patients were reported from Chile [69] and Brazil [12], respectively. In the first case, the nematode was not identified to the species level, but a recent study reported the presence of microfilariae morphologically and phylogenetically related to *D. repens* in dogs from Chile [13]. This represents the first report of a *Dirofilaria* species in Chile and suggests a late introduction of this parasite in this country, which until recently was considered as a *Dirofilaria*-free area [4,45,70]. Further studies are advisable to better characterize this parasite. Similarly, a recent case of ocular dirofilariosis (Figure 2) was reported in a 16-year-old boy from Pará State, northern Brazil [12]. The parasite was morphologically and phylogenetically very similar to *D. immitis*. However, high nucleotide differences (5% and 6% for 12S rDNA and *cytochrome c oxidase subunit 1*, respectively) was found by comparing sequences from the nematode recovered from the patient’s eye with sequences obtained from dogs living in the same area and/or from other countries available in GenBank [12]. This case report suggested that different strains or cryptic species of *Dirofilaria*, which is close to *D. immitis*, might be circulating in Brazil and in the western hemisphere as a whole.

### A more virulent ‘*D. immitis’* in the Americas?

A critical analysis of human dirofilariosis cases attributed to *D. immitis* in the Old World concluded that there is no proof demonstrating that Old World *D. immitis* plays a pathogenic role in humans [8]. Indeed, human pulmonary dirofilariosis in the Old World appears to be always associated with *D. repens*, even if *D. immitis* is more prevalent than *D. repens* in both dogs and vectors in some areas [42]. Based on this critical appraisal of human dirofilariosis in the Old World, two main hypotheses were proposed to explain this intriguing situation [8]. First, there could be different *D. immits* genotypes in the New and Old Worlds, with varying infective capacities for dogs and humans. Alternatively, some unidentified factor, probably related to the vector, could modify the infective capacity of the parasite to humans in the Old World. Although both hypotheses are plausible and deserve to be investigated more in-depth, there is no evidence indicating the existence of a more virulent *D. immitis* strain in the Americas.

Even if some individuals can present with cough, chest pain, haemoptysis, and dyspnoea [62,63,67,71], the majority of the cases of human pulmonary dirofilariosis reported in the literature refer to asymptomatic individuals that present a solitary, well-circumscribed, non-calcified peripheral subpleural pulmonary nodule (“coin lesion”), which are usually located in the lower lobes [62,72]. These nodules may mimic a malignant tumour and are often found by chance on chest radiograph and chest computerized tomography scans, which are usually requested for other reasons [62]. Importantly, in most cases, the identification of the parasite species is based on histological findings, which may not be adequate [8]. In fact, alterations in the parasite structures or the degeneration of worms inside nodules [4] may impair the identification of species.

## Conclusions

Considering the usually benign nature of *D. immitis* infestation in human hosts, most cases of pulmonary dirofilariosis will likely remain without a definitive diagnosis. As a consequence, the actual number of pulmonary dirofilariosis in the Americas is likely to be grossly underestimated at present. Nonetheless, the existence of a more virulent strain of *D. immitis* in the Americas remains uncertain. So far, available data do not support this hypothesis, mainly considering that most human patients present no apparent clinical signs.

There are several lacunae in our knowledge regarding different aspects of animal and human dirofilarioses in the Americas. For example, *bona fide* information about the species of filarioids infesting dogs and humans in this region is meagre. Further studies are needed to better characterize filarioids circulating among different domestic and wild animals in the American continent. Certainly, the use of an integrated genetic (e.g., DNA barcoding using mitochondrial genes) and morphological approach could be beneficial for the identification of filarioids [73].

The high diversity of potential zoonotic dirofilariae in the Americas is undisputed, but information on the biology of most wildlife-associated *Dirofilaria* species is currently lacking. Similarly, scientific knowledge on the biology of *Dirofilaria* species infestation in humans is limited for obvious ethical reasons and several aspects of the host-parasite interactions (e.g., the proportion of inoculated larvae that develop, microfilariae migration routes) remain unknown. Certainly, studies on bacterial endosymbionts associated with *Dirofilaria* nematodes may provide further data on their biology and evolution [74], also considering that the presence or absence of *Wolbachia pipientis* in *Dirofilaria* species has been associated with the immuno-pathology of dirofilariosis [4,74]. In particular, dogs, cats, and humans may develop strong IgG response for the dominant *Wolbachia* surface protein and the participation of this bacterium in inflammatory processes occurring during dirofilariosis has been intensively investigated in recent years [4]. In addition, tetracyclines targeting the *Wolbachia* endosymbionts of filariae were useful in damaging or even killing *D. immitis* adult worms [75]. Therefore, the combination of doxycycline and ivermectin in long-lasting administration, in the place of melarsomine injections, succeeded in eliminating adults of *D. immitis*, eventually reducing the risk of thromboembolism [76]. A similar, therapeutic protocol, applied monthly, was shown to be effective for treating microfilariaemia in dogs affected by subcutaneous dirofilariosis by *D. repens*[77,78].

The competence of different culicid species/strains in the Americas as vectors of *Dirofilaria* species should be better investigated. Such studies would help us to understand possible variations in transmission patterns and even to predict possible scenarios that may emerge in the future, with the introduction of non-endemic species/strains in free areas through importation of infested animals, vectors, or both. In this context, mathematical models and distribution maps are extremely important for predicting the presence/absence and abundance of mosquito vectors in different regions.

Finally, it is crucial to increase awareness among veterinary practitioners and medical physicians regarding the zoonotic significance of filarial nematodes of domestic and wild animals in the Americas. This is particularly important in remote areas, such as the Amazon region, where a different range of zoonotic, yet unknown filariae [79] is likely present.

## Competing interests

The authors declare there are no conflicts of interest.

## Authors’ contributions

FD-T wrote the manuscript and DO reviewed critically it. Both authors approved the final version of the manuscript.
